# Transhepatic access closure for islet cell transplant in anticoagulated patients: a comparison of microfibrillar collagen paste, coils, and coil plus gel foam

**DOI:** 10.1186/s42155-025-00623-1

**Published:** 2025-12-09

**Authors:** Qian Yu, Patrick Tran, Ethan Ungchusri, Kunal Karani, Abdul Khan, Mikin Patel, Osman Ahmed, Thuong Van Ha, Jonathan Lorenz, Steven Zangan, Brian Funaki, Rakesh Navuluri

**Affiliations:** Department of Radiology, Vascular and Interventional Radiology, University of Chicago Medical Center, University of Chicago, Chicago, IL 60637 USA

**Keywords:** Embolization, Islet cell transplant, Portal vein, Transhepatic access

## Abstract

**Purpose:**

To evaluate the safety and effectiveness of microfibrillar collagen paste (MCP), coils, and coils combined with gelatin sponge for transhepatic access tract embolization following portal vein islet cell transplant.

**Methods:**

A retrospective review was conducted at a single institution between January 2008 and October 2024, including 20, 28, and 21 consecutive islet cell transplant procedures requiring transhepatic access embolization with MCP, coils, and coil plus gelatin sponge, respectively. All procedures were performed via a right portal vein branch. MCP was performed using Avitene (BD). The average number of coils required in the coil plus gelatin sponge and coil-only groups were 1.8 and 1.6 coils per procedure, respectively. All patients were placed on therapeutic anticoagulation during the procedure and for at least two weeks post-transplant. Medical records were reviewed to compare laboratory results, portal venous pressures, post-procedure liver ultrasounds, and 30-day hemorrhagic events across the three groups.

**Results:**

All procedures were technically successful. However, one instance of coil migration into a portal vein branch occurred in the coil plus gelatin sponge group (1/28, 3.5%). Baseline hemoglobin, platelet counts, and partial thromboplastin time did not differ significantly between groups (*p* > 0.05). A statistically significant lower international normalized ratio (INR) was observed in the MCP group compared to the gelatin sponge and coil-only groups (1.0 vs. 1.1 vs. 1.1, p = 0.0036 and 0.004). No statistically significant differences were found in hemoglobin changes, post-transplant portal venous pressures, or post-embolization hemorrhagic events (*p* > 0.05). One patient in the coil plus gelatin sponge group developed a large subcapsular hematoma (1/27, 3.7%), while another in the MCP group experienced a large right hemothorax (1/20, 5.0%).

**Conclusion:**

MCP, coils, and coil plus gelatin sponge are similarly effective for transhepatic access closure following islet cell transplant in anticoagulated patients. However, coil embolization may require multiple coils and carries a risk of migration.

## Introduction

Managing type 1 diabetes mellitus (T1DM) can be challenging, as insulin therapy effectively controls blood glucose levels but is not curative, and sporadic, potentially fatal hypoglycemic episodes may still occur [1]. Islet cell transplantation has been shown to achieve insulin independence in some patients, and advances over the last two decades in deriving islet cells from pluripotent stem cells have expanded the availability of this procedure [2]. Interventional radiology plays a critical role in islet cell transplantation, as cells are injected percutaneously into the portal veins [3, 4]. After islet cell infusion, the transhepatic access tract is embolized to reduce the risk of hemorrhage, given that these patients require therapeutic anticoagulation to prevent portal vein thrombosis. Previous study has suggested that microfibrillar collagen paste (MCP) may offer advantages over gelatin sponge for achieving hemostasis at the access site following islet cell transplantation [5]. Other options, including coils, vascular plugs, liquid embolics, and various combinations of embolic materials, are also effective options in closing transhepatic access tracts in other interventional radiology procedures [6]. Among these, pushable coils are relatively cost-effective, though their efficacy compared to MCP remains uncertain. This study aims to compare the safety and effectiveness of MCP, coils alone, and coils combined with gelatin sponge for transhepatic access tract embolization following islet cell transplantation.

## Materials and methods

A single institutional retrospective review was performed to include consecutive adult patients who underwent percutaneous portal vein islet cell transplantation from June 2008 to October 2024. A total of 69 patients who underwent tract embolization using either MCP, coils only, or a combination of coils and gelatin sponge were included in the study (mean age 42.9 ± 9.1 years; 41 out of 69 patients were female, representing 59.4%). The number of patients who underwent embolization with MCP, coils, and a combination of coils and gelatin sponge were 20, 28, and 21, respectively.

### Percutaneous transhepatic islet cell transplant

**Fig. 1:**
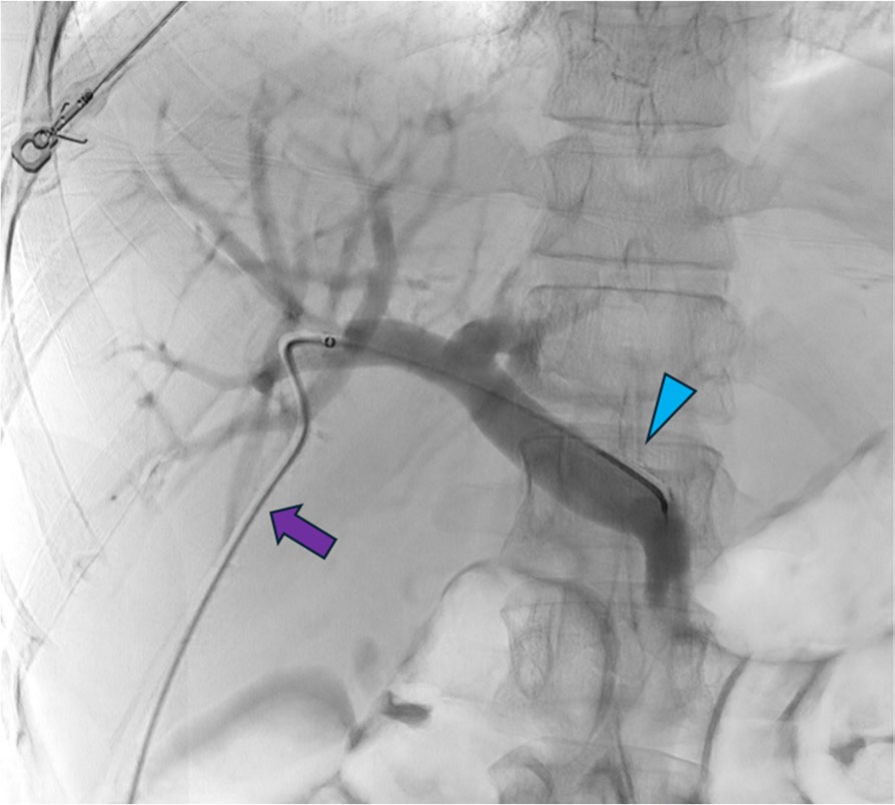
Tranhepatic portal venogram via a 6 French Accustick sheath (purple arrow) and 4 French Kumpe catheter (blue arrowhead) coaxial system

Percutaneous transhepatic islet cell transplant was performed under conscious sedation and local anesthesia as previously described^3^. In brief, the right portal vein branch was accessed with a 21-gauge Accustick (Boston Scientific, Natick, MA, USA) or Chiba needle (Cook, Bloomington, IN, USA) under real-time ultrasound guidance. An 0.018 guidewire was advanced into the portal vein. Over the wire, a 6 Fr AccuStick sheath (Boston Scientific, Natick, MA, USA) was placed into the main portal vein and a portal venogram was obtained. In some cases, a 4 Fr Kumpe catheter was used to access the main portal vein through the 6 Fr Accustick sheath in a co-axial fashion (Fig. 1), depending on operator preference and if there was difficulty advancing the sheath forward.

Intraprocedural systemic anticoagulation, islet cell collection and administration methods were described previously [7]. Pre-procedure and post-procedural portal vein pressures were measured using a transducer. After the procedure, systemic intravenous heparin continued throughout the hospital stay. Serial hemoglobin and coagulation labs were obtained. Abdominal ultrasound was performed on post-procedural day one to assess portal vein patency and hematoma. Patients were bridged to therapeutic subcutaneous enoxaparin upon discharge.

### Tract embolization

After islet cell administration, the embolic was deployed through the catheter just peripheral to the portal vein puncture and as the sheath was retracted to the capsule. The sheath was removed. Manual pressure was held as the puncture site. Post procedural ultrasound images were performed to evaluate placement of the embolic and evidence of hemorrhage. A sterile bandage was applied to the puncture site.

**Fig. 2:**
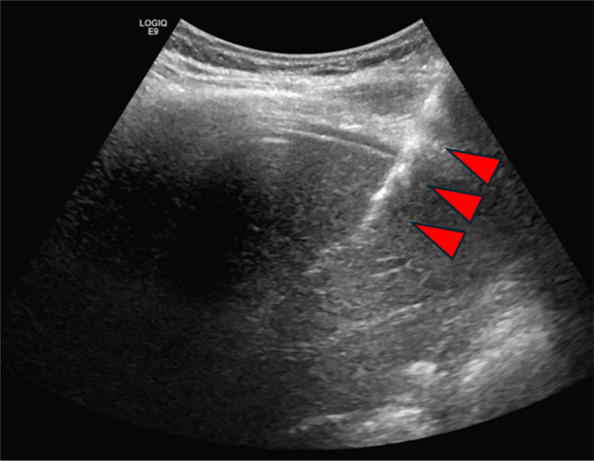
Linear echogenicity (red arrow heads) from the microfibrillar paste injected within the transhepatic access tract

Microfibrillar collagen paste was prepared using one package of Avitene (BD, Franklin Lakes, NJ, USA), which was in powder form, mixed with 6 mL saline using a three-way stopcock and two syringes. The paste was injected using the access sheath under ultrasound guidance (Fig. 2). For coil embolization, 0.035″ coils were deployed through the 4 Fr Kumpe catheter under fluoroscopic guidance. A variety of coils were deployed at operator’s preference, including Interlock (Boston Scientific Corporation, Natick, MA, USA), Nester (Cook Medical, Bloomington, IN, USA), and MReye® Embolization Coil (Cook Medical, Bloomington, IN, USA). In general, a 4 mm coil was deployed initially, and the use of additional coils was at the discretion of the operator, especially when more central access resulted in a longer tract. When a combination of gelatin sponge and coils were used, gelatin sponge slurry or torpedo was injected through the access sheath under ultrasound guidance after coil embolization. Gelatin slurry was prepared by mixing saline and EmboCube (Merit Medical, South Jourdan, UT, USA), or gelatin sponge sheet cut into stripes; injection was performed under real time ultrasound guidance with care not to over inject into the portal vein. Torpedoes were prepared by cutting a gelatin sponge sheet into stripes as a previous study had described [5]. Iodinated contrast was not used after islet cell infusion.

### Statistical analysis

Data were summarized as mean ± standard deviation (SD) for numerical variables and as counts and percentages for categorical variables. Baseline characteristics and outcomes across tract embolization groups were compared using t-tests for continuous variables and chi-square or Fisher’s exact tests for categorical variables. A p-value of < 0.05 was considered statistically significant. All statistical analyses were conducted using Stata 18.0 (StataCorp, College Station, TX, USA).

## Results

### Comparison of baseline characteristics

There was no significant difference among patient age, sex, and prior islet cell transplant history among three groups. Baseline hemoglobin, platelet counts, and partial thromboplastin time did not differ significantly between groups (*p* > 0.05). Compared to the gelatin sponge and coil-only groups, the MCP group demonstrated lower baseline INR (p = 0.004), but this difference was not clinically significant (1.0 vs 1.1 vs 1.1, *p* > 0.05).

### Technical complications

**Fig. 3:**
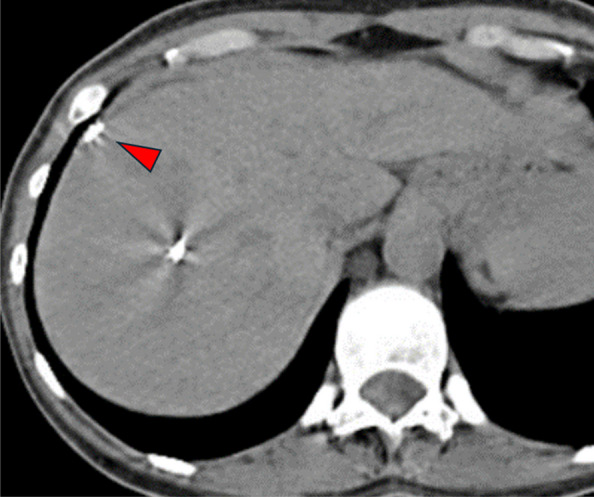
Coil migration after embolization on computed tomography (arrowhead)

**Fig. 4:**
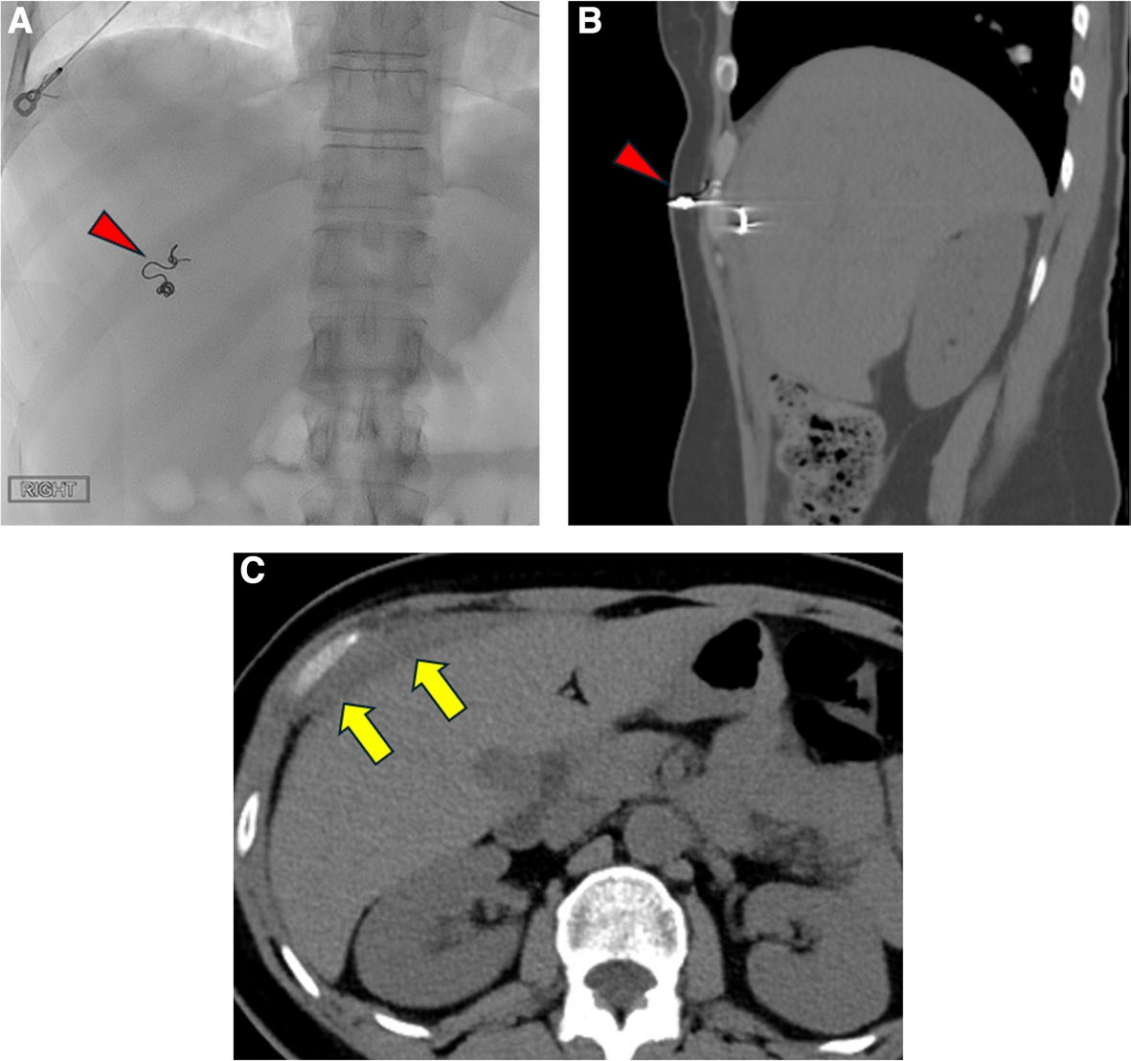
Coil mis-deployment resulting in half of the coil being extrahepatic. **A** fluoroscopic image showing misdeployed coil (red arrowhead). **B** sagittal computed tomography (CT) showing the coil partially situating in the subcutaneous tissue (red arrowhead). **C** axial CT showing a small subcapsular hematoma (yellow arrows)

No more than one package of MCP was used. The average number of coils required in the coil plus gelatin sponge and coil-only groups were 1.8 and 1.6 coils per procedure, respectively. All procedures were technically successful. One instance of coil migration into a portal vein branch occurred in the coil plus gelatin sponge group (1/28, 3.5%, Fig. 3). In the coil-only group, one instance of mis-deployment occurred, when the pushable coil displaced the Kumpe catheter and access sheath external to the patient body, resulting in half of the coil free-floating in the extrahepatic space (1/21, 4.8%, Fig. 4). The coil caused persistent local tenderness, which was then removed under local anesthesia in the clinic two weeks after the transplant. No statistically significant differences were found in post-transplant portal venous pressures, or changes in portal venous pressures (*p* > 0.05).

### Hemorrhagic complications

**Fig. 5:**
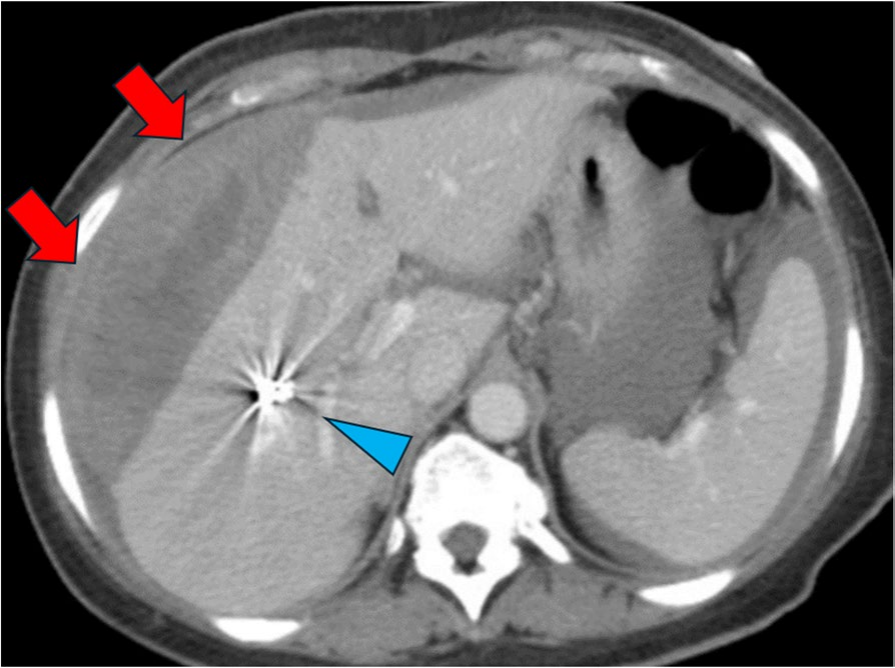
Computed tomography showed subcapsular hematoma (red arrows) after islet cell transplant. Blue arrowhead: coils

**Fig. 6:**
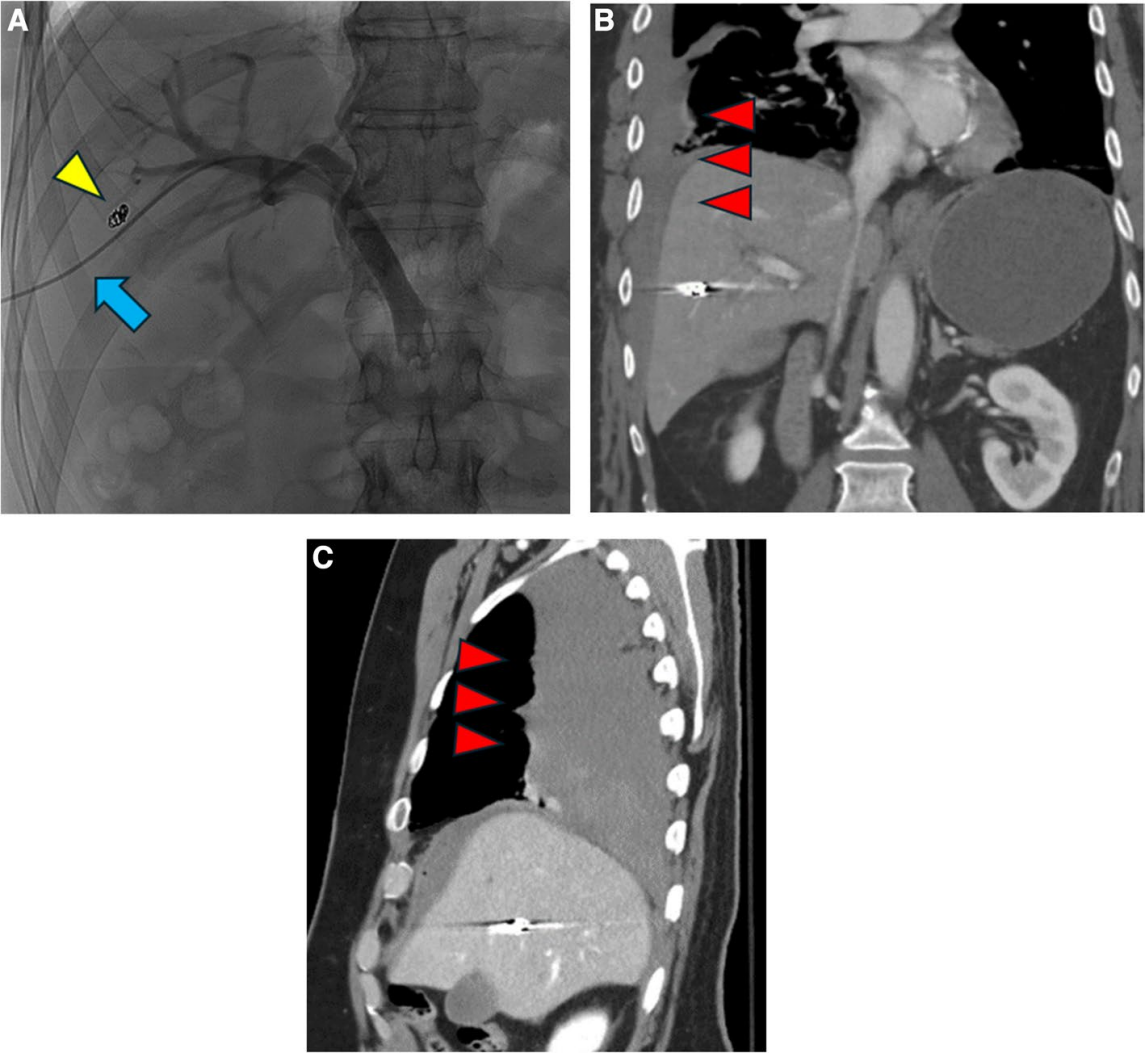
Right hemothorax after islet cell transplant via intercostal approach. **A** fluoroscopy demonstrated the intercostal approach transhepatic access (blue arrow: access sheath; yellow arrowhead: existing coil from prior islet cell transplant). **B** coronal and **C** sagittal computed tomography demonstrated right hemothorax (red arrowheads)

After patients started on heparin drip, the mean overall post-transplant PTT was 67.5 ± 46.0, which were similar among three groups (Table 1). No statistically significant differences were found in post procedural hemoglobin and changes in hemoglobin (*p* > 0.05). One patient in the coil plus gelatin sponge group developed a large subcapsular hematoma (1/28, 3.75, Fig. 5), while another in the MCP group experienced a large right hemothorax (1/20, 5.0%, Fig. 6); both cases were managed with transfusion and holding of anticoagulation The patient who had coil-misdeployment from the coil-only group as described above also had a small perihepatic hematoma (1/21, 4.8%, Fig. 4). The rates of post-embolization hemorrhagic events were not statistically significantly different among the three groups.

**Table 1:** Baseline characteristics and outcomes

Variables	Microfibrillar Collagen Paste (n = 20)	Coil + gel foam (n = 28)	Coil-only (n = 21)	P value (Microfibrillar Collagen Paste vs Coil + Gelfoam)	P value (Microfibrillar Collagen Paste vs Coil-only)	P value (Coil + Gelfoam vs Coil-only)
Age (years)	44.5 (9.0)	43.2 (9.8)	41.0 (8.8)	0.659	0.207	0.402
Sex Male Female	8 (40.0%) 12 (60.0%)	10 (35.7%) 18 (64.3%)	11 (56.4%) 10 (47.6%)	0.762	0.623	0.401
Hemoglobin (g/dL) Pre Post Change	12.0 (1.9) 10.9 (1.8) 1.1 (0.8)	11.8 (1.6) 10.9 (1.6) 0.91 (0.93)	12.2 (2.2) 11.1 (2.2) 1.0 (0.77)	0.708 0.960 0.403	0.785 0.685 0.755	0.496 0.675 0.576
Platelet count (10^3/mL)	251.8 (81.1)	234.3 (115.3)	235.1 (59.2)	0.564	0.457	0.975
Preprocedural PTT (s)	28.5 (2.9)	29.2 (3.6)	28.5 (2.6)	0.506	0.996	0.487
Preprocedural INR	0.99 (0.075)	1.1 (0.087)	1.1 (0.075)	**0.0036**	**0.0004**	0.764
Transplant number Initial Subsequent	14 (70.0%) 6 (30.0%)	14 (50.0%) 14 (50.0%)	10 (47.6%) 11 (52.4%)	0.166	0.146	0.869
PVP (mmHg) Pre Post Change	6.4 (3.5) 8.0 (3.5) 1.6 (1.1)	8.9 (4.7) 10.2 (5.0) 1.2 (2.0)	8.3 (3.3) 9.3 (3.3) 1.5 (2.5)	0.0528 0.089 0.516	0.0773 0.220 0.873	0.663 0.478 0.732
Postprocedural PTT(s)	66.6 (10.0)	68.1 (47.9)	50.8 (13.5)	0.915	0.128	0.115
Bleeding event	1 (5.0%)	1 (3.6%)	1 (4.8%)	0.807	0.972	0.835

*INR *international normalized ratio, *PTT *partial thrombin time, *PVP *portal vein pressure

## Discussion

This study demonstrated that transhepatic closure with MCP was as durable as coils, with or without additional gelatin sponge embolization, following islet cell transplant. No significant differences were observed among the three groups in post-procedural hemoglobin levels or hemoglobin changes. Additionally, the three embolization methods had similar risks of major hemorrhage, with one case occurring in each group. In the MCP group, the one case of postprocedural hemorrhage was likely due to puncture through the intercostal artery rather than insufficient hemostasis from the embolic material itself. In contrast, the two hemorrhagic cases in the coil groups were related to inadequate hemostasis of the transhepatic tract, leading to subcapsular hematoma.

From a technical standpoint, coil use carried a risk of misdeployment if not carefully managed, which occurred twice in this study. While one instance of coil migration was clinically insignificant, the other led to local tenderness and a small subcapsular hematoma, ultimately necessitating coil removal. From a cost-effectiveness perspective, MCP is a low-cost embolic that can be easily prepared at the back table. Compared to gelatin sponge, MCP can activate the coagulation pathway more effectively to achieve hemostasis, proving superior to gelatin sponge alone for tract closure in islet cell transplant [5, 8, 9]. In contrast, multiple coils were typically needed for tract closure, making this approach more costly than MCP, even with the use of pushable coils. Although newer generations of detachable coils could reduce the risk of misdeployment, their cost is substantially higher than MCP or pushable coils.

Previous studies have demonstrated that tract embolization following islet cell transplantation is associated with a reduced risk of bleeding, so it is recommended for transhepatic islet cell transplantation [6, 10]. Tract embolization may also be indicated for other transhepatic procedures, including liver biopsy and portal venous interventions [11–13]. In addition to gelatin sponge, microfibrillar collagen paste, and coils, other embolic agents such as vascular plugs and n-butyl cyanoacrylate glue have been utilized [6, 14, 15]. However, these latter devices are associated with substantially higher costs in the authors’ practice setting and therefore may not be cost-effective for routine application. The results of this study may be applicable to other transhepatic interventions, such as portal vein recanalization, mesenteric vein thrombolysis, and portomesenteric variceal embolization^6^. However, comparing closure methods in these procedures can be challenging due to variability in patient characteristics, intervention types, and sheath sizes. In aforementioned settings, patient populations often include individuals with cirrhosis and impaired coagulation, whereas patients undergoing islet cell transplants require therapeutic anticoagulation. In all of these scenarios, there is an increased risk of hemorrhagic events. Thus, MCP, as a cost-effective option for tract closure in access sizes up to 6 Fr, is likely suitable for transhepatic interventions beyond islet cell transplantation.

This study should be interpreted with several limitations. First, the small sample size and single-institution retrospective design may limit statistical significance. Additionally, other closure methods, such as glue and vascular plugs, were not evaluated despite their growing popularity in transhepatic interventions. Compared to MCP and coils, liquid embolic such as glues might obviate the requirement of intact coagulation cascade, and thus be more effective in the setting of islet cell transplant [6]. Also, the radiopacity of these embolics could also improve real-time visualization under fluoroscopy during deployment. Furthermore, as noted, it remains unclear whether newer generations of detachable coils offer a higher technical success rate in this setting [16].

In conclusion, MCP and coils with or without gelatin sponge were equally effective in tract closure after islet cell transplant. Whereas coils carry the risk of misdeployment, MCP appears to be a more cost-effective option.

## Data Availability

Upon request.
